# Fast Active Merging of Microdroplets in Microfluidic Chambers Driven by Photo-Isomerisation of Azobenzene Based Surfactants

**DOI:** 10.3390/bios9040129

**Published:** 2019-11-01

**Authors:** Zain Hayat, Nizar Bchellaoui, Claire Deo, Rémi Métivier, Nicolas Bogliotti, Juan Xie, Malcolm Buckle, Abdel I. El Abed

**Affiliations:** 1 Laboratoire de Photonique Quantique et Moléculaire (LPQM), UMR 8537, Ecole Normale Supérieure Paris Saclay, CentraleSupélec, CNRS, Université Paris-Saclay, 61 avenue du Président Wilson, 94235 Cachan, France; ZAIN.HAYAT@ens-paris-saclay.fr (Z.H.); NIZAR.BCHELLAOUI@ens-paris-saclay.fr (N.B.); 2 Photophysique et Photochimie Supramoléculaires et Macromoléculaires (PPSM), UMR 8531, Ecole Normale Supérieure Paris Saclay, CNRS, Université Paris-Saclay, 61 avenue du Président Wilson, 94235 Cachan, France; claire.deo@embl.de (C.D.); Remi.METIVIER@ppsm.ens-cachan.fr (R.M.); NICOLAS.BOGLIOTTI@ens-paris-saclay.fr (N.B.); joanne.xie@ens-paris-saclay.fr (J.X.); 3 Laboratoire de Biologie et Pharmacologie AppliquéE (LBPA), UMR 8113, Ecole Normale Supérieure Paris Saclay, CNRS, Université Paris-Saclay, 61 avenue du Président Wilson, 94235 Cachan, France; buckle@ens-paris-saclay.fr

**Keywords:** microdroplets, photo-isomerisation, photokinetics, opto-mechanics, conformational states

## Abstract

In this work, we report on the development of a newly synthesized photoactive reversible azobenzene derived surfactant polymer, which enables active and fast control of the merging of microdroplets in microfluidic chambers, driven by a pulsed UV laser optical stimulus and the well known *cis*-*trans* photo-isomerisation of azobenzene groups. We show for the first time that merging of microdroplets can be achieved optically based on a photo-isomerization process with a high spatio-temporal resolution. Our results show that the physical process lying behind the merging of microdroplets is not driven by a change in surface activity of the droplet stabilizing surfactant under UV illumination (as originally expected), and they suggest an original mechanism for the merging of droplets based on the well-known opto-mechanical motion of azobenzene molecules triggered by light irradiation.

## 1. Introduction

Many lab-on-a-chip (LoC) applications have become possible thanks to the ability to control mixing of different droplet contents, which enabled the sequencing of many complex bio-chemical and biological reactions with a high level of control and flexibility over the last decade; see for a review [1,2,3,4,5,6,7]. Hence, among all manipulation schemes allowed by droplet-based microfluidics technology [8,9,10,11,12,13,14,15,16], active merging of microdroplets (AMD) is probably one of the most important. It is generally achieved using a high alternating current (AC) voltage [17], or using a direct current (DC) voltage [18]. Nevertheless, light-driven merging of droplets is a more attractive approach since light provides not only high temporal and spatial resolutions but also wavelength and intensity tunability [19,20,21].

**Figure 1:**
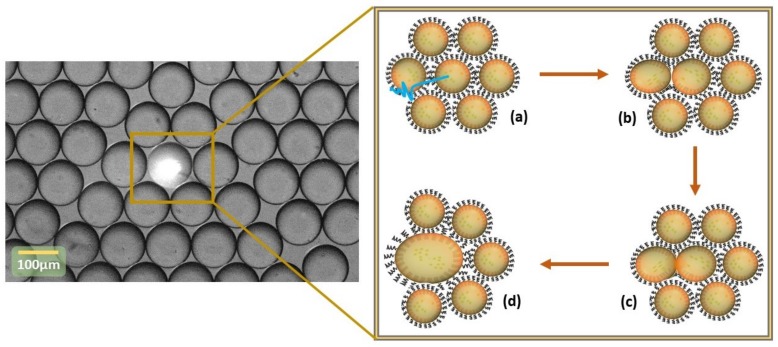
Light-driven merging process principle. (**a**) Targeting droplets and change of surface activity of the surfactant molecules at the droplet surface under laser irradiation, (**b**) depletion of the surfactant molecules at the the droplet interface, (**c**) merging induced following UV laser irradiation and the *trans* to *cis* photo-conversion, (**d**) targeted droplets after merging.

**Figure 2:**
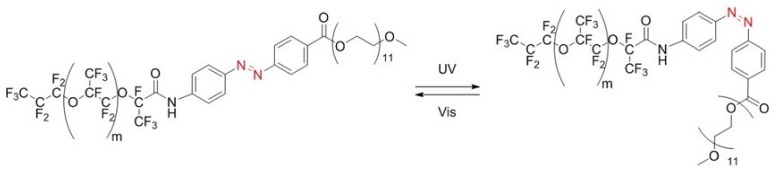
Stable *trans* form (**left**) and metastable *cis* form (**right**) of the synthesized KryAz600 molecule; *trans* to *cis* transition occurs under UV irradiation and back to *trans* occurs under visible light.

Recently, Dunkel et al. [22] showed that active merging of microdroplets can be achieved optically and very selectively using the photolysis process of photolabile surfactants [22]. In the present study, we consider a new strategy based on the photo-isomerization process (Figure 1) of a newly synthesized azobenzene derivative surfactant polymer, whose structure is given in Figure 2 and which is named in this study KryAz600, to achieve an active merging of water-in-oil (W/O) microdroplets using a picosecond (ps) pulsed UV laser.

This study was inspired initially by a previous work carried out by Takahashi et al. [23], who reported that a light-induced destabilization of an overall emulsion based on the photo-isomerization process of azobenzene-derived photosensitive surfactants, through the light induced interfacial activity change of gemini-like azobenzene derived surfactants and the conversion between a higher surface activity *trans* isomer and a lower surface activity *cis* isomer. This resulted in a destabilisation of the overall emulsion without the aim of achieving spatial differentiation and requesting several minutes of irradiation.

Our approach is different and is far from trivial. In fact, the dynamics of the change of azobenzene surfactants surface activity at the microscale, at which new interfaces are produced involve both the change of surface tension driven by the photo-isomerisation process and the diffusion of the new surfactant molecules to the interface, as well as the adsorption on the droplet surface, each partial step adding a typical time and length-scale [24]. Our approach is also different from the recent study reported by Dunkel et al. [22] as the mechanisms lying behind the two merging processes involved in the two studies are completely different. Moreover, photo-isomerization of azobenzene is fully reversible, which makes this new approach particularly suitable for the reuse of the photo-sensitive surfactant, which is generally produced in a small quantity.

## 2. Materials and Methods

### 2.1. Chemicals

Azobenzene derived molecules possess two stable geometric isomers: an energetically stable *trans* form and a meta-stable *cis* form. For most azobenzenes, molecules can be optically isomerized from *trans* to *cis* using light in the near UV and Visible: upon absorption of a photon with a wavelength in the near UV (around 330 nm), molecules convert, with high efficiency, from the *trans* isomer into the *cis* isomer. A second photon with a wavelength in the visible range (around 440 nm) can induce the back-conversion. UV illumination can also enable conversion of azobenzene molecules from a *cis* form to a *trans* form as photons of UV light have higher energy than visible light, which is sufficient to induce *cis* to *trans* isomerization. Azobenzene photo-isomerization is completely reversible and both forward and reverse photoisomerizations typically exhibit picosecond timescales. The *trans* isomer is thermodynamically more stable than the *cis* isomer, by approximately 50–100 kJ/mol and the energy barrier for thermal isomerization is in the order of 100–150 kJ/mol. Hence, in the dark, the *cis* isomer thermally relaxes back to the *trans* isomer on a timescale ranging from milliseconds to hours, or even days, depending on the substitution pattern around the azobenzene group and the local environment of the molecules.

We synthesized a new fluorinated azobenzene derivative surfactant polymer, named in this study KryAz600. It consists of a triblock copolymer surfactant, composed of a perfluoro-polyether (PFPE) hydrophobic chain, linked to a polyethylene-glycol (PEG-600) hydrophilic chain (Sigma-Aldrich, Saint-Quentin Fallavier, France) through an azobenzene group, as shown in Figure 2. The PFPE hydrophobic chain was derived from a commercially available carboxy-terminated fluorinated polymer, namely Krytox 157-FSH (Dupont) and linked to the azobenzene group following a similar procedure as described in detail by Lee et al. [25], see also supporting information at (S1) for details.

### 2.2. Experimental Setup

**Figure 3:**
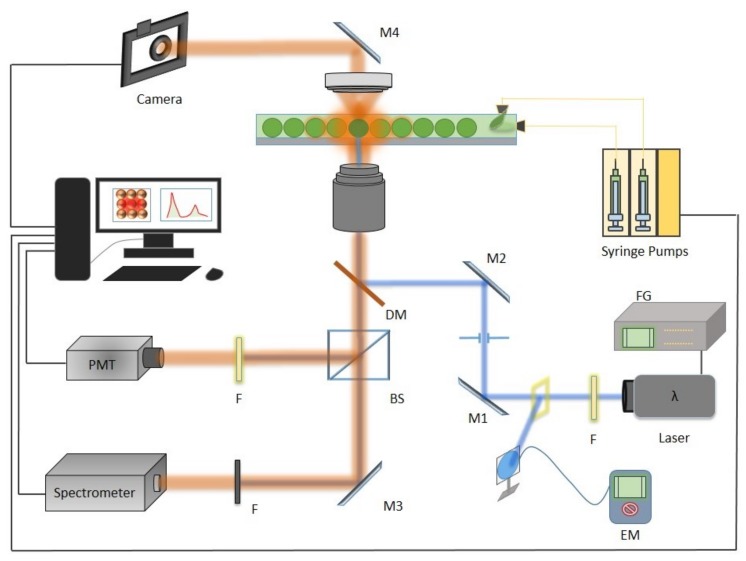
The setup containing the droplet generation assembly, optics and detection unit. On the optical path, the ps-pulsed source (355 nm), F (filter), M1 and M2 mirrors for step-up, dichoric mirror (DM) (506 nm), camera with notch filter, BS (beam splitter) to reflect the acquired signal to detector (setup adapted from [15]).

Nemesys syringe pumps (Cetoni GmbH, Korbussen, Germany) were used to fabricate monodisperse drops of size range 50 μm to 150 μm depending upon the need. To study and sort droplets, the optics part consists of a ps-pulsed UV laser source, with a peak wavelength at 355 nm and delivering 15.4 μJ of energy per pulse. In order to make the laser spot size adequate, the laser beam was cleaned and band-limited for enhanced detection (20 nm filter F). Figure 3 illustrates the optical path for the laser source. After the filter, the source was staged up and reflected by dichroic mirror (DM) (Tx = 506 nm, Semrock, Rochester, NY, USA) to the sample where a microscope objective (2×, 4×, or 10× Olympus Inverted Microscope) targets and acquires the reflected/scattered signal from the droplet under observation. At the detection side we used a standard speed camera for recording and visual inspection of the droplet generation, manipulation, and merging. For detection, a photo-multiplier tube (Hamamatsu) was used to monitor droplet generation frequency. For optimized 100 μm drop-size (500 μL/h and 100 μL/h for the continuous and dispersed phase), the droplet frequency was found to be about 250 drops/second (scheme for droplet frequency was derived from the method discussed in [15]).

### 2.3. Design and Microfabrication

Droplets were generated in a flow-focusing microfluidic device, which was fabricated using the standard soft-lithography technique, employing PDMS (Polydimethylsiloxane) for replica molding. The constructed microfluidic device had a square drive channel (guide length after the drop-maker) and a rectangular micro-analysis chamber constructed by stepper lithographic pattern. The advantage of the dual stage chamber is to facilitate the droplet in maintaining a spherical shape, which in turn reduced the pressure coalescence of microdroplets. Droplet stability can be increased by providing extra time to the surfactant molecule in order to localize around the droplet (exterior) wall. This attribute was achieved by increasing the drive channel length to almost three times the length of the drive channel mentioned in previous work [22].

**Figure 4:**
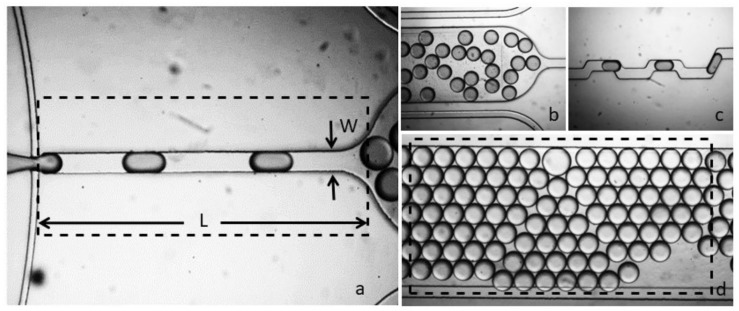
The fabricated flow-focusing assembly, (**a**) drive channel with width W and length L, (**b**) droplet generation at 500 uL/h and 100 uL/h for drive and dispersed phase, (**c**) output channel for droplet frequency monitoring, (**d**) micro-analysis and storage chamber.

The drive channel length was set to about 2100 μm (Figure 4a), which is long enough to allow the oil soluble surfactant molecules to build a stabilizing monolayer around the droplets before the droplet come in contact with each other at the entrance, where they may merge. After the drive channel, droplets enter a rectangular chamber of the dimensions 4000 × 1200 μm (Figure 4b) acting as droplet storage and an analysis chamber. At the end of the device assembly, a zig-zag channel leads to output for the droplet collection (Figure 4c). The advantage of droplet collection is to utilize the reversibility of the photo-active reversible compound and to perform off-chip micro-particle studies.

In order to make the device interior compatible with the oil-phase, the interior walls of the complete assembly was functionalized by commercially available surface coating agent, which consisted of a 2% solution of perfluoroctyl-dimethylsiloxane dissolved in HFE7100 fluorocarbon oil (3M). This coating enhanced the wettability of the channel walls and also reduced the risk of diffusion of the surfactant molecules to the PDMS. For the droplet generation, we used a fluorinated oil phase (HFE 7500, 3M, density 1.62 g/cm${}^{3}$).

Photochromic reactions were induced in-situ by a continuous irradiation Hg/Xe lamp (Hamamatsu, LC6 Lightningcure, 200 W) equipped with narrow band interference filters of appropriate wavelengths. The irradiation power was measured using a photodiode from Ophir (PD300-UV). The photochromic quantum yields were determined by probing the sample with a Xenon lamp during the course of the light irradiation. Absorption changes were monitored by a charge coupled device (CCD) camera mounted on a spectrometer (Ocean Optics, Largo, FL, USA).

## 3. Results and Discussion

### 3.1. KryAz600 Surfactant Photokinetics

**Figure 5:**
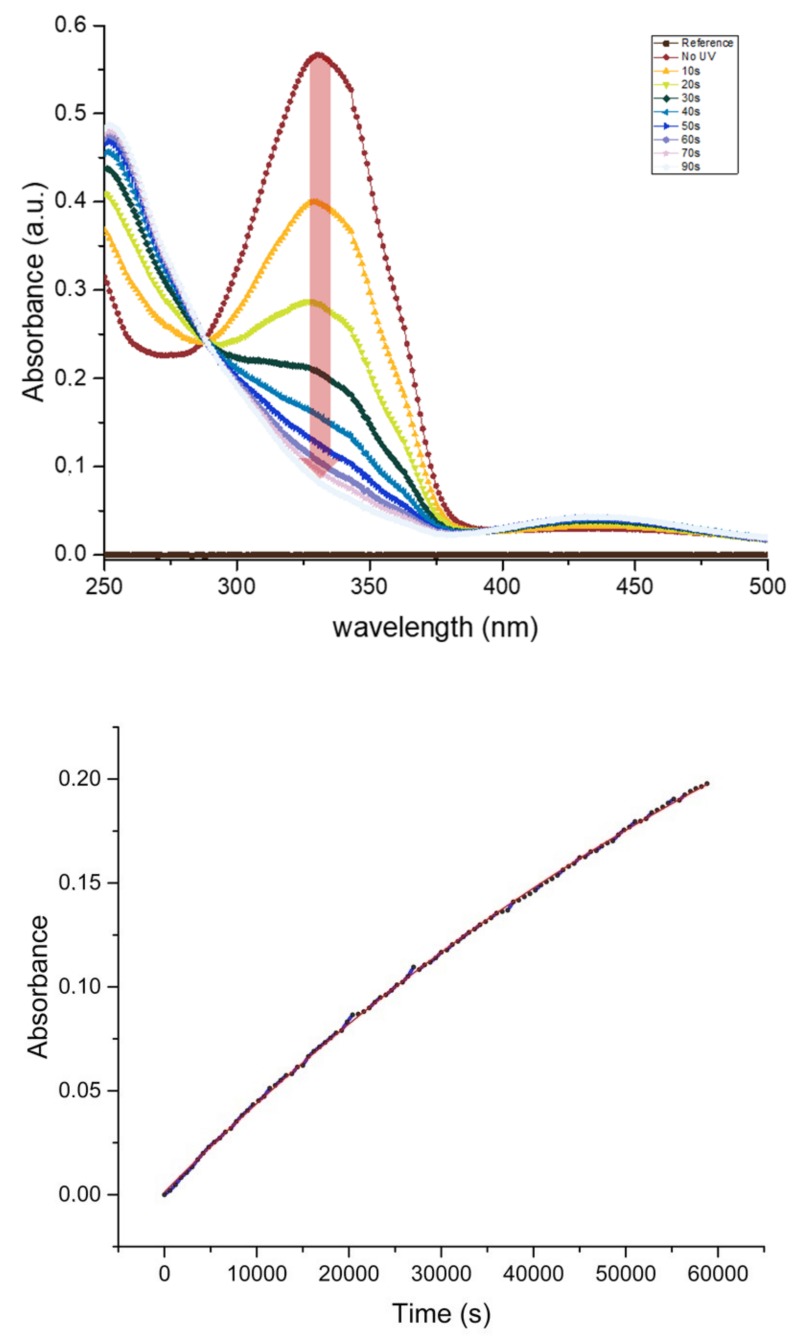
(**Upper**) Conversion of the surfactant state under UV illumination, Top curve (red) being the first measurement with no UV treatment and subsequent state change transition of surfactant by 10 s of exposure to a UV lamp with power of 6.7 mW at 365 nm wavelength; (**Bottom**) slow exponential rise of the 335 nm absorption band intensity of the t-KryAz600 form in dark, this corresponds to a half-life $t_{1/2}≃18$ h of the c-KryAz600 → t-KryAz600 transition.

Likewise, most azobenzene derivatives, KryAz600 molecules switch under UV illumination from a stable *trans* form (t-KryAz600) to a metastable *cis* form (c-KryAz600). The *trans* form is characterized by a large absorption band with a maximum absorption around 335 nm (in HFE 7500 oil), while c-KryAz600 form has a weaker absorption band with a maximum absorption around 440 nm, as shown in Figure 5.

Our results show that KryAz600 molecules transit spontaneously in the dark from the *cis* form to the *trans* form with a constant time as small as $10^{-5}$/s. This value was deduced from the exponential fit of the absorption curve of c-KryAz600 at 330 nm versus time, as shown in Figure 5. It corresponds to a half-life $t_{1/2}≃18$ h. It is worth noting that the relatively long half-life of KryAz600 molecules is of great importance in our study since the *cis* form relaxes back very slowly to the *trans* form, if no UV illumination is used.

### 3.2. Microdroplets Stability Versus Surfactant Conformation and Concentration

Monodisperse microdroplets with a size of about 100 μm were prepared using different surfactant concentrations dissolved in HFE7500 oil, ranging from 1 mM down to 15 μM.

In order to quantify the effect of the *cis* and *trans* conformations on the stability of droplets, a control experiment was first conducted. Two samples of equal concentration of 1 mM ($C_{0}$) were prepared, one was left in the dark (overnight) in order to allow for all surfactant molecules present in the solution to transit to the thermodynamically stable *trans* state, and the other sample was illuminated with a 235 nm UV lamp for 1 h.

**Figure 6:**
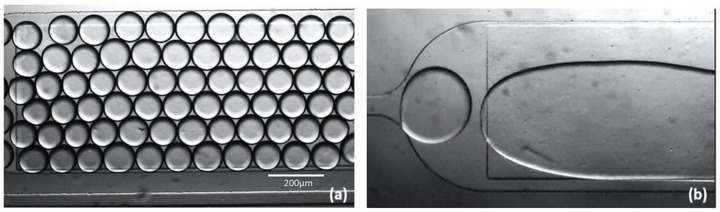
Surfactant KryAz600, (**a**) mono-disperse (water-in-oil) droplets of stable *trans* state, (**b**) effect of UV exposure, unstable *cis* state causing coalescence of micro-droplets.

Droplets that were prepared using the non irradiated surfactant solution exhibited long term stability (Figure 6a) where as the droplets prepared using UV illuminated surfactant solution merged immediately in the observation chamber (Figure 6b). This shows that *cis* conformation of KryAz600 molecules is not suitable to ensure droplet stabilization. A quick glance at the molecule structure of Figure 2 shows that the *cis* conformation is less suitable to achieve a close packing of surfactant molecules at the droplet interface, than could be done using the *trans* isomer. In other words, *cis* isomer molecules lead to a lower surface density of surfactant molecules around the droplet and hence to a higher interfacial tension.

It is worth noting, that UV illuminated surfactant solution, when left in the dark overnight, enables us again to produce stable droplets. This demonstrates the reversibility of the process and the possibility to reuse surfactant solution for further experiments. Our observations show also that the merging of microdroplets under laser illumination is not the result of the thermo-capillary effect of the laser beam, since thermal effects are present for both droplets, prepared with the two types of isomers.

Also, because at the micrometer scale, the diffusion of surfactant molecules cannot be neglected, we found it necessary to investigate the relation between the merging time and the concentration of the surfactant molecules in the carrier fluorocarbon oil. This is particularly important in our study since a focused pulsed laser with a micrometer sized footprint is used for photo-isomerization.

Water-in-oil (W/O)droplets were produced and collected in an on-chip micro-analysis chamber with different surfactant concentrations, decreasing from 1 mM ($C_{0}$) to 15.6 μM. This process depends on the kinetics of the depletion of surfactants under laser illumination. The first concentration, $C_{0}=1$ mM of the surfactant, was found to lead to very stable microdroplets with no merging process with irradiation times smaller than 10 min, whereas a fast merging (∼1 s) could be achieved with a concentration value of 25 μM. It is worth noting that for smaller concentration values of surfactant (*c* < 25 μM), a spontaneous merging of droplets is observed, which indicates that there are not enough surfactant molecules in the carrier oil solution for droplets stabilization. Successive dilutions of the surfactant solution resulted in a decrease of the merging time from about 10 min for $C_{0}$ (1 mM) to 1 s for 25 μM. For lower concentrations, droplets were observed to be highly unstable. These results clearly indicate a decrease in merging time as the concentration of surfactant molecules decreases. Merging reduced sufficiently from almost 6 s for 62.5 μM to 1 s for 15.6 μM. This result may be interpreted as follows. As the concentration reduces, the number of idle-molecules in bulk drive phase take more time to replace the targeted molecules. Also, since the surfactant molecules are not oriented in a specific order, so the lower the concentration, the higher the diffusion time for the idle molecules to reach the depleted area.

To better understand these results, let us first calculate the flow time, $t_{flow}$, that each droplet takes to reach the observation micorchamber; $t_{flow}$ corresponds to the flight time of droplets along the drive channel, from the nozzle to the end of the main channel, above which droplets start to collide with each other. Considering the length L=2100μm of the drive channel and the mean velocity of microdroplets in this region, v≃30 mm/s, one finds:

(1) $$t_{flow}=\frac{L}{v_{flow}}≃70\;\mathrm{ms}.$$

To ensure droplet stability, $t_{flow}$ should be greater than the diffusion time, $t_{dif}$, which is approximately the necessary time lapse for building a stabilizing surfactant layer around the droplets to prevent their coalescence. Indeed, during $t_{flow}$, a given number of surfactant molecules diffuse from the bulk phase and adsorb arround the flowing droplet interface to form a stabilizing surfactant layer. Considering the total area $S_{drop}=4\pi R^{2}$ of a microdroplet with a radius *R* (≃50 μm), the maximum packing of surfactant molecules, with a typical lateral dimension δ∼2 nm, i.e., with a molecular area $A∼\delta^{2}$, at the interface of the droplet is achieved with *n* molecules when $4\pi R^{2}∼n\delta^{2}$. Droplets will be stable when the number of molecules at the interface is larger than a fraction 0<f<1 of the maximum packing; therefore, the stability condition reads

(2) $$n\geq \frac{4f\pi R^{2}}{A}.$$

According to Baret et al., [26] microdroplets become stable when the number of molecules at the interface becomes larger than a fraction f∼0.1 of the maximum packing; therefore, the stability condition for microdroplets of radius *R* can be expressed as follows:

(3) $$n∼\frac{0.4\pi R^{2}}{A}\mathrm{molecules}.$$

The number of free surfactant molecules required for the stabilization of the droplet interface, *n*, can be assumed to be dispersed in a volume $V_{\epsilon }=4\pi R^{2}\epsilon$ surrounding the droplet over a distance ϵ from the interface (ϵ<<R). Hence, *n* can be estimated as $n∼4\pi R^{2}\epsilon cN_{A}$, where $N_{A}$ is Avogadro’s number. In a diffusion limited process, surfactant molecules confined in the volume $V_{\epsilon }$ will reach the droplet interface within a time $t_{dif}$ given by the diffusion law: $\epsilon^{2}∼D\times t_{dif}$. It is possible thus to estimate an experimental value for the diffusion time $t_{dif}$ according to the following equation:

(4) $$t_{dif}∼\frac{0.01}{{\left(N_{A}cA\right)}^{2}D}.$$

Hence, for c=25 μM, one finds $t_{dif}∼30$ ms ($<t_{flow})$, whereas for *c* = 15 μM, one finds $t_{dif}∼70$ ms ($≃t_{flow}$). These results are in good agreement with the observed surfactant concentration threshold for droplet stability, which was found to lay in the 25–15 μM range.

**Figure 7:**
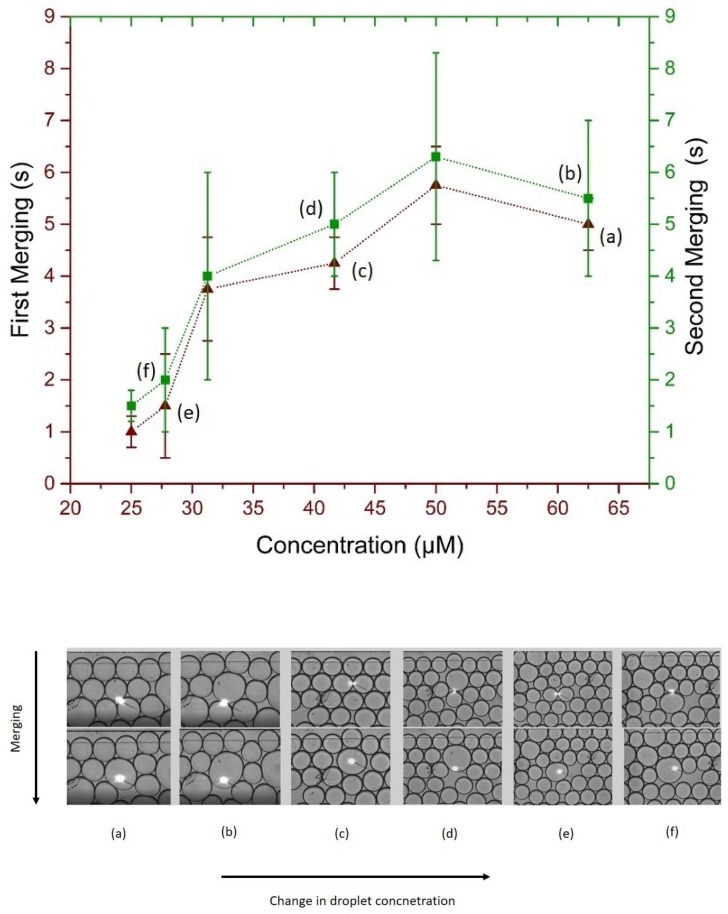
(**Upper**) Merging time scale of irradiated droplets, maroon (triangles) for first merging and green (squares) for second merging, (**Bottom**) optical micro-graphs of the first and second merging and resulting change in targeted droplet volume.

To further analyze the merging behavior of microdroplets under irradiation, different sweeps of the laser beam over the whole micro-chamber were performed and first and second merging of the one drop to its neighbor and later (second merging) with the two times big merged drop to its neighboring droplet. Results presented in Figure 7 show a non-uniform but correlated first and second merging times. The rationale behind two merging observations relates to the change in volume and a slight increase in the merging time. Consider Figure 7, on left, two drops selected for merging and on right the big merged drop. For two separate drops of 100 μm, the calculated volume was 0.5 nL each, while after merging the the big drop had a volume of 4 nL, thus eight times increase in volume. From the classical inverse relation of concentration with volume, the overall change in concentration of big merged drop was 0.125 times the concentration of the drive phase. Upon targeted merging of that droplet with the usual droplet caused a slight increase in merging time, depicted as a green curve in Figure 7.

### 3.3. Suggested Opto-Mechanical Model for Droplet Merging Mechanism

Unlike in the work reported by Takahashi et al. [23], where a broad UV illumination during several minutes of the overall emulsion resulted in a destabilisation of the overall emulsion, photo-isomerization is achieved in our study using a ps UV laser which is focused on a very tiny fraction of the emulsion. It lasts only a few seconds before droplet merging is achieved. At such time and space scales, the change in the activity of surfactant molecules involves both a change of surface tension driven by the photo-isomerisation process and the diffusion of new surfactant molecules from the bulk solution to the interface, as well as the adsorption on the droplet surface. If one considers a diffusion limited merging process mechanism, to achieve droplet interface destabilization and merging, the rate of photoisomerized surfactant molecules under pulsed UV laser illumination should be higher than the diffusion rate of non irradiated surfactant molecules from the bulk solution to the target droplet interface. Nevertheless, in the particular case of photo-isomerization, one should take account of the fact that the higher energy UV illumination can induce not only isomerization from *trans* to *cis* form but it can also induce the reverse transformation on *cis* molecules present under the UV light spot. Hence, when a pulsed UV laser is used, if one illuminates locally, for instance, a population of A isomers during a first pulse to B isomers, then the transformed B isomers would go back to their initial conformation (A) under illumination during the following pulse, unless diffusion is fast enough to deplete the illuminated region of B isomers during the first pulse of the UV laser.

Though UV light can drive both *cis* to *trans* and *trans* to *cis* photo-isomerizations, one should emphasize that a longer term UV illumination of azobenzene molecules would not lead in average to a mixture of 50% of molecules in each state. Indeed, the forward (*trans* to *cis*) and backward (*cis* to *trans*) photo-reactions of azobenzene molecules do not show the same quantum yield, i.e., the probability of inducing the photo-reaction after absorption of a photon. Therefore, the ratio of *trans* and *cis* isomers upon long term UV irradiation can be much higher than 50%. In the present case, this ratio is higher than 85% under 365 nm illumination as can be deduced from the absorbance curves of KryAz600 molecules around 335 nm after 90 s of illumination vs. their absorbance before irradiation (0 s), as shown in Figure 5.

Let us calculate the diffusion distance ϵ of surfactant molecules in the oil solution during the time lapse $\tau_{pulse}$ of a first single pulse, which is equal to 1 ms in our experiments (and which should not be confused with the duration of a pulse ≃ 400 ps). Using diffusion law, and considering a typical value of the diffusion coefficient of surfactant molecules in HFE7500 oil, about $D≃10^{-10}$ m${}^{2}$/s [26,27], one finds a mean diffusion distance of

(5) $$\epsilon ∼\sqrt{D\tau_{pulse}}∼0.3\;\mu m,$$

During the time lapse of one pulse. Even if considering a diffusion coefficient which is two orders of magnitude higher than the previous one, as reported by Dunkel et al. [22], one would find a mean diffusion distance of about ϵ∼3 μm during each pulse. It is interesting to note that such values are much smaller than the size of the illuminated area with the focused laser beam, which is, ∼30 μm in our experiments. This rough estimation of the diffusion of surfactant molecules during the time lapse between two successive illuminations shows that a large proportion of isomerized molecules during the first pulse will be still present in the illuminated area when the following light pulse triggers and hence *cis* isomers will recover their initial conformation (*trans*) under this second UV laser pulse.

Consequently, one should conclude that the observed merging process of droplets cannot be attributed to a change of surface activity of surfactant molecules under illumination, as surfactant molecules switch continuously from *trans* to *cis* and from *cis* to *trans* under successive UV laser pulses. The merging process should be based on opto-mechanical effects due to the well-known change of the length of the *cis* and *trans* azobenzene groups under illumination. Photo-isomerization reduces the distance between the 4 and 4’ positions of azobenzene groups from 0.99 nm in the *trans* state to 0.55 nm for the *cis* state [28]. Consequently, UV laser illumination produces geometric changes in the azobenzene group, which should translate into larger-scale motions extending to the overall surfactant droplet interface layer. After approximately one second of illumination, the oscillation of the droplet interface should lead to the merging of the droplets.

## 4. Conclusions

Active merging of microdroplets is one of the most important manipulations enabled by droplet-microfluidics, as it enables for on demand droplet targeting and sequencing of complex chemical and biological reactions. Nevertheless, the standard voltage-based methods are generally limited to spatially isolated pairs of droplets, as the long-range effect of the applied electric field does not have the potential to differentiate between flowing or confined droplets. We developed an optofluidic approach, which overcomes such limitations and allows for a rapid controlled coalescence of targeted microdroplets thanks to the application of a ps UV pulsed laser. Our approach is based on the photo-isomerization process of an azobenzene-derived surfactant. The success of such controlled coalescence is far from trivial. In fact, the dynamics of the change of surface tension at the microscale driven by photo-isomerisation processes; the diffusion of the new surfactant molecules to the interface; as well as the adsorption on the droplet surface, are complex and not well understood. By incorporating light activated switching in the surfactant, release or mixing of droplets contents could be programmed. In this study, we present the fast and dynamic switching of azobenzene derived surfactant for controlled coalescence of spatially selectable microdroplets as proof of concept. 

## Supplementary Materials

The following are available online at https://www.mdpi.com/2079-6374/9/4/129/s1, Figure S1: Chemical synthesis procedure of KryAz600 surfactant molecules.
