# Supplementary material for: Fast Active Merging of Microdroplets in Microfluidic Chambers Driven by Photo-Isomerisation of Azobenzene Based Surfactants
Source: Biosensors (Basel). 2019 Nov 1;9(4):129. doi: 10.3390/bios9040129 (PMC6956313; doi:10.3390/bios9040129)
Supplement: Supplementary file 1 [file biosensors-09-00129-s001.pdf]

Supporting information (S1) : Chemical synthesis procedure of KryAz600 surfactant.

General Procedures

All purchased reagents were used without further purification. HFE7100 was dried on molecular sieves 4Å. ^{1}H and ^{13}C NMR spectra were recorded on a JEOL 400 spectrometer and referenced to the resonance of the solvent used. Infrared (IR) spectra were recorded with a Nicolet Nexus FT-IR spectrometer equipped with an ATR-Germanium unit and are reported as wavenumbers (cm^{-1}). High-resolution mass spectra (HRMS) were performed on a Bruker maXis mass spectrometer by the "Fédération de Recherche" ICOA/CBM (FR2708) platform.

Synthetic scheme

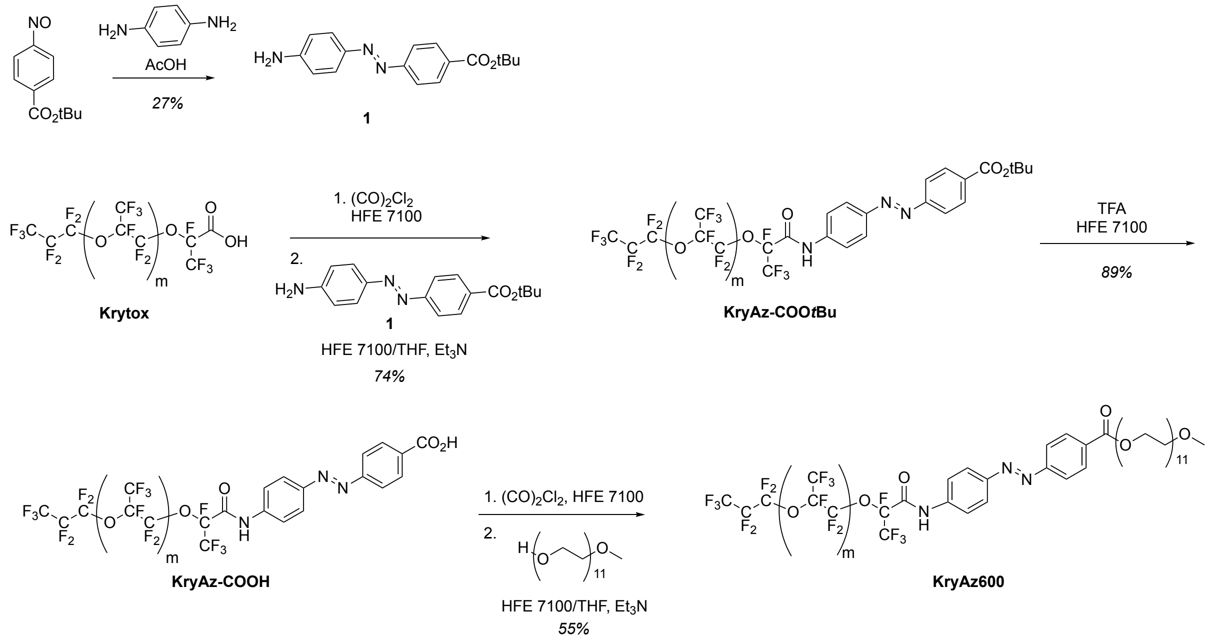

Synthetic procedures

1: To a solution of *t*-butyl 4-nitrosobenzoate (95 mg, 0.46 mmol) in AcOH (4 mL) was added 4-phenylenediamine (49 mg, 0.45 mmol, 1 equiv). After stirring at 60°C for 12 h, the mixture was evaporated under reduced pressure. The residue was dissolved in CH_2Cl_2/H_2O (1/1, 50 mL) and the layers were separated. The aqueous layer was extracted with CH_2Cl_2 (3 x 10 mL), the combined organic layers were washed with a saturated aqueous solution of $NaHCO_3$, dried over anhydrous Na_2SO_4, filtered and evaporated under reduced pressure. The crude product was purified by column chromatography (silica gel-PE/EA: 7/3) to afford **1** (37 mg, 0.12 mmol, 27%) as an orange solid; ^{1}H NMR (400 MHz, $CDCl_3$): δ ppm 8.10 (d, *J* = 8.2 Hz, 2H), 7.84 (m, 4H), 6.75 (d, *J* = 8.7 Hz, 2H), 4.14 (br s, 2H, NH_2), 1.63 (s, 9H); ^{13}C NMR (100 MHz, $CDCl_3$): δ ppm 165.6, 155.5, 150.3, 145.7, 132.7, 130.5, 125.7, 122.1, 114.7, 81.4, 28.4; HRMS (ESI): calc. for $C_{17}H_{20}N_3O_2^+$, $[M+H]^+$ *m/z* 298.1550, found *m/z* 298.1550.

KryAz-COO*t*Bu: To a solution of Krytox® (2.22 g, 0.32 mmol) in anhydrous HFE7100 (7 mL) under argon was added oxalyl chloride (0.2 mL, 10 equiv). After stirring at 60°C for 12 h, the mixture was evaporated under reduced pressure. To a solution of the crude product in anhydrous HFE7100 (8 mL) under argon was added a solution of **1** (95 mg, 0.32 mmol, 1 equiv) and Et_3N (0.2 mL) in anhydrous THF (8 mL). After stirring at r.t. for 12 h, the supernatant was removed and the orange oil was washed with THF (3 x 15 mL) to afford **KryAz-COO*t*Bu** (1.62 g, 0.24 mmol, 74%) as an orange oil. **IR (ATR):** 1705, 1693 cm^{-1}.

KryAz-COOH: To a solution of **KryAz-COO*t*Bu** (1.62 g, 0.24 mmol) in HFE7100 (5 mL) was added trifluoroacetic acid (100 µL, 1.35 mmol, 5.6 equiv). After stirring at r.t. for 12 h, the mixture was evaporated under reduced pressure. The crude product was washed with H_2O (4 x 5 mL), acetone (2 x 5 mL) and CH_2Cl_2 (2 x 5 mL) to afford **KryAz-COOH** (1.43 g, 0.21 mmol, 89%) as an orange oil. **IR (ATR):** 1784, 1708, 1690 cm^{-1}.

KryAz600: To a solution of **KryAz-COOH** (930 mg, 0.138 mmol) in anhydrous HFE7100 (6 mL) under argon was added oxalyl chloride (225 µL, 1.38 mmol, 10 equiv). After stirring at 60°C for 12 h, the mixture was evaporated under reduced pressure. The crude residue in solution in anhydrous HFE7100 (6 mL) was added to a solution of *O*-methyl-undecaethylene glycol (80 mg, 0.15 mmol, 1.2 equiv) and Et_3N (37 µL, 0.28 mmol, 2 equiv) in anhydrous THF (6 mL). After stirring at r.t. for 12 h, the supernatant was removed and the yellow oil was washed with H_2O (2 x 5 mL) acetone (2 x 5 mL) and CH_2Cl_2 (2 x 50 mL) to afford **KryAz600** (920 mg, 0.127 mmol, 92%) as a yellow oil. **IR (ATR):** 1707, 1693 cm^{-1}.

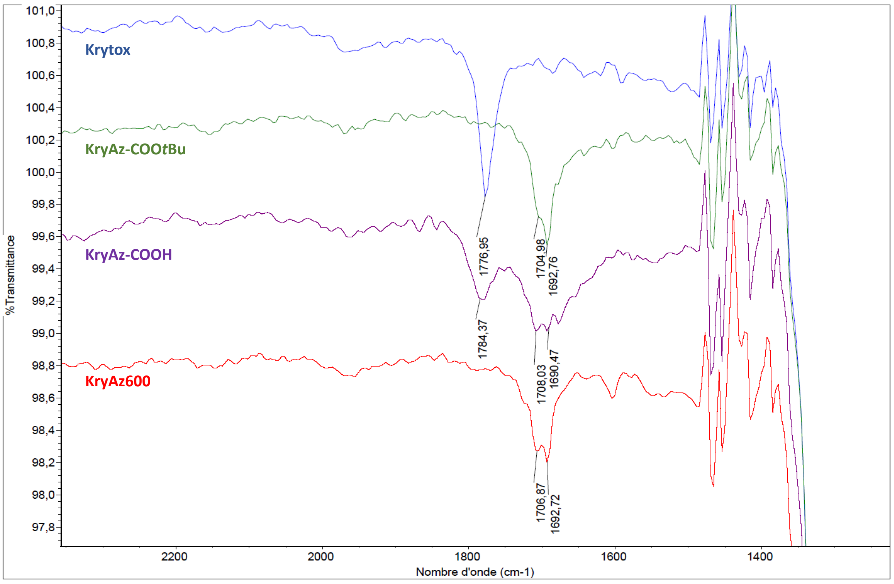

Figure S1. Portion of the infrared spectra of the different surfactants synthesized.
